# Economic Hardship and Violence: A Comparison of County-Level Economic Measures in the Prediction of Violence-Related Injury

**DOI:** 10.1177/08862605221118966

**Published:** 2022-08-29

**Authors:** N. Jeanie Santaularia, Marizen R. Ramirez, Theresa L. Osypuk, Susan M. Mason

**Affiliations:** 1 University of Minnesota School of Public Health, Minneapolis, USA; 2 University of Minnesota, Minnesota Population Center, Minneapolis, USA

**Keywords:** violent injury, child abuse, intimate partner violence, elder abuse, economic hardship, foreclosure

## Abstract

Economic hardship may lead to a wide range of negative outcomes, including violence. However, existing literature on economic hardship and violence is limited by reliance on official reports of violence and conflation of different measures of economic hardship. The goals of this study are to measure how violence-related injuries are associated with five measures of county-level economic shocks: unemployment rate, male mass layoffs, female mass layoffs, foreclosure rate, and unemployment rate change, measured cross-sectionally and by a 1-year lag. This study measures three subtypes of violence outcomes (child abuse, elder abuse, and intimate partner violence). Yearly county-level data were obtained on violence-related injuries and economic measures from 2005 to 2012 for all 87 counties in Minnesota. Negative binomial models were run regressing the case counts of each violence outcome at the county-year level on each economic indicator modeled individually, with population denominator offsets to yield incidence rate ratios. Crude models were run first, then county-level socio-demographic variables and year were added to each model, and finally fully-adjusted models were run including all socio-demographic variables plus all economic indicators simultaneously. In the fully-adjusted models, a county’s higher foreclosure rate is the strongest and most consistently associated with an increase in all violence subtypes. Unemployment rate is the second strongest and most consistent economic risk factor for all violence subtypes. Lastly, there appears to be an impact of gender specific to economic impacts on child abuse; specifically, male mass-lay-offs were associated with increased rates while female mass-lay-offs were associated with decreased rates. Understanding the associations of different types of economic hardship with a range of violence outcomes can aid in developing more holistic prevention and intervention efforts.

## Introduction

Economic hardship, or the difficulty caused by having too little money or too few resources, is a social determinant of health. Factors that comprise economic hardship include but are not limited to challenges with employment, affordable housing, and access to transportation. Economic hardship may lead to a wide range of devastating impacts, including decreased use of preventive and primary care services (Addington, 1999; Ayanian, 2000; Zuvekas & Taliaferro, 2003), increased risk for hospitalization for chronic health conditions (Addington, 1999; Ayanian, 2000), and increased probability of financial debt creating a snowball effect throughout life (Cutshaw et al., 2016). The purpose of this analysis was to examine how different types of county-level economic hardships are related to three types of violence: child abuse, intimate partner violence (IPV), and elder abuse. Below we review the literature on existing evidence and provide a theoretical framework for the study.

### Economic Hardship and Violence

Substantial evidence suggests that one potential outcome of economic hardship is violence. A recent systematic review found evidence of an association between the foreclosure crisis in 2008 with both violent crime and child abuse (Downing, 2016). Another systematic review examined different measures of economic hardship (including debt, poverty, material hardship, income, income losses, unemployment, housing hardship, etc.) and their impacts on child abuse and found varying effects depending on the economic measure. Results indicated that housing hardships are most strongly related to childhood abuse, while poverty showed mixed associations (Conrad-Hiebner & Byram, 2020). In addition, there is a relatively large amount of literature looking at the effects of other macro-economic conditions on a range of violence outcomes including child maltreatment (Conrad-Hiebner & Byram, 2020; Coulton et al., 1995; Deccio et al., 1994; Drake & Pandey, 1996; Freisthler et al., 2007; Frioux et al., 2014; Gillham et al., 1998; Pare, 1992; Paxson & Waldfogel, 2002; Steinberg et al., 1981; Tobey et al., 2013; Wood et al., 2012; Young & Gately, 1988; Zuravin, 1986), IPV (Aizer, 2010; Alvira-Hammond et al., 2014; Cho & Kim, 2012; Copp et al., 2016; Crowne et al., 2011; Cunradi et al., 2002; Edwards, 2015; Fox et al., 2002; Franklin & Menaker, 2014; Golden et al., 2013; Heise, 1998; Litton Fox & Benson, 1974; Sabina, 2013; Showalter, 2016; VanderEnde et al., 2015), and violent crime (Rosenfeld, 2009, 2014). For example, several studies have found that foreclosure rates (Frioux et al., 2014), neighborhood poverty (Drake & Pandey, 1996), and higher county level income inequality (Eckenrode et al., 2014) are associated with an increase in child maltreatment. Furthermore, male employment (Edwards, 2015), city-level unemployment (Heise, 1998), and the gender wage gap (Aizer, 2010) are risk factors for IPV. Lastly, studies have found that a higher US regional level unemployment rate (Rosenfeld, 2009) and several city, county, and economic indicators (Baumer et al., 2018) are related to increased homicide.

### Theoretical Framework

The current literature suggests that different types of economic measures may operate differently with respect to violence because they capture conceptually different economic hardship concerns. For example, social disorganization theory suggests that foreclosure may contribute to community violence through increased residential mobility and subsequent lack of social cohesion (Armstead et al., 2021). This lack of social cohesion may, in turn, reduce a community’s power to control violence and also remove social supports during times of hardship. Alternatively, strain theory or the family stress model suggests that unemployment may put undue strain on a family’s financial resources, increasing the risk for child maltreatment through family stress (Agnew, 1999; Conger & Donnellan, 2007). Whether one of these pathways is more important than the other and whether this differs depending on the type of violence remains unknown. These theories illustrate the importance of considering multiple types of economic hardship measures that may operate in different ways. However, existing studies often operationalize economic hardship through single measures, making it difficult to directly compare which measures are most strongly associated with violence. Another reason that different aspects of economic hardship may have different impacts is that they may be early (unemployment) or lagging (foreclosure) indicators of financial hardship and may be more or less addressable with existing resources (e.g., unemployment impacts may be mitigated by unemployment insurance [UI]).

### Measurement of Violence

While there is existing research examining the association between economic hardship and violence, these studies have relied on violence surveillance data that have important limitations. Specifically, traditional violence surveillance systems, such as those operated by government agencies (law enforcement or child protection data) may suffer from inherent selection bias defined as “distortions that result from procedures used to select subjects and from factors that influence study participation” (Rothman et al., 2008) and/or misclassification defined as “bias in estimating an effect. . .caused by measurement errors in the needed information” (Rothman et al., 2008). Selection bias is of concern due to historic over-reporting and over-policing in marginalized and highly scrutinized communities (e.g., poor children have more interaction with mandated reporters through public benefits programs) (Bor et al., 2018; Maguire-Jack et al., 2018; Putnam-Hornstein et al., 2013; Thurston & Miyamoto, 2020). Misclassification is also a potential problem in these data because of systemic under-identification of violence in certain populations, which may contribute to biased estimates. Alternative data sources may therefore be important for advancing understanding of the patterns and determinants of violence.

One possible alternative or complementary data source for surveillance and research on violence is hospital discharge data on violence-related injuries. Unlike data from law enforcement or child protection, which may over-represent violence in certain subsets of the population, hospital discharge data capture anyone who comes into the hospital to seek treatment for a severe physical injury. Specifically, injuries known to be caused by violence are captured through standardized International Classification of Disease (ICD) codes. (e.g., ICD-9: 995.81 for child physical abuse) (Scott et al., 2009). There are, however, notable limitations to the use of these explicit violence codes. In particular, they are underutilized and potentially biased, because patients must reveal that the injury that brought them into the hospital was due to violence, or the provider must make a subjective assessment that this was the case. Thus, codes that explicitly diagnose violence-related injuries may be prone to similar underreporting or misreporting as in other surveillance systems like crime data. One potential way to address this challenge is through the use of proxy codes to supplement violence identification (R. P. Berger et al., 2011; Santaularia et al., 2021). These proxy codes consist of ICD codes that identify injuries not explicitly indicated as violence but that are highly correlated with violence. Using these proxy codes for violence identification as a complement to explicit violence injury codes may yield a more holistic view of the distribution and determinants of violence in the population.

### Current Study

In summary, the current literature has two main gaps when assessing the economic hardship–violence association. First, economic hardship has tended to be operationalized through a single measure, despite the possibility that various types of economic hardship may influence violence differently. Second, there is potentially underreporting and/or misreporting of violence in common violence surveillance systems that could lead to biased estimates of associations. To address these gaps, the current study assesses the association of a range of indicators of economic hardship with rates of violence-related injuries derived from hospital discharge data. Specifically, the goals of this study are to measure how violence-related injuries are associated with nine measures of county-level economic hardship: unemployment rate, male mass layoffs, female mass layoffs, foreclosure rate, and unemployment rate change. This study measures three subtypes of violence (child abuse, elder abuse, and IPV) through explicit diagnostic codes (injuries where the cause is identified as intentional). To address concerns about under- or biased use of explicit violence codes, additional analyses utilizing proxy codes (injuries likely due to violence where the cause is not explicitly identified as intentional) are also conducted. Based on social disorganization theory (Armstead et al., 2021), strain theory (Agnew, 1999), and prior research (Lindo et al., 2018), we hypothesize that each county economic hardship measure will be positively associated with both county rates of proxy and explicit codes for all violence subtypes, with the exception of female-mass layoffs, which will be negatively associated with each violence subtype. In addition, we hypothesize that county economic hardship measures will have stronger associations with explicit measures of violence compared to proxy measures of violence, reflecting a potential skew of the explicit measures toward poorer and marginalized populations.

## Methods

This ecologic study merges yearly county-level observations from 2005 to 2012 from multiple sources (described below) for all 87 counties in Minnesota. These years of data are used because of the availability of several economic hardship measures.

### Data

#### Minnesota hospital discharge data

A population-based hospital administrative data set containing a census of hospital visits was obtained through the Minnesota Hospital Association (MHA) from 2005 to 2012. Hospitals in the State of Minnesota submit inpatient, outpatient, and emergency department claims data to the MHA. The MHA collects these data into a statewide administrative claims database. This database includes a data point for each patient encounter with a health care provider and includes the diagnosis/es (ICD codes) during that encounter, as well as basic patient demographic information, such as age and gender.

There are three categories of diagnostic codes in hospital claims data. ICD-9 codes describe the diagnosis of the condition and/or the treatment, and are required for billing. E-codes and V-codes are modifiers to ICD-9 codes that provide additional detail, but they are not required. In the case of injuries, E-codes describe when and where the injury happened, to whom or by whom, and how. V-codes, also known as history codes, provide information about the history of the diagnosis. Neither E-codes nor V-codes are required for billing (*ICD-9-CM—International Classification of Diseases, Ninth Revision, Clinical Modification*, 2019, p. 9). Repeated annual cross-sections of data on ICD-9, E-codes, and V-codes are used to measure cases of violence for this study. The ways each of these codes are used to assess violence is described in more detail in the variable operationalization sections below.

#### Economic data

All annual county-level economic data used here are publicly available. Specifically, unemployment and mass layoff data are from the U.S. Bureau of Labor Statistics (BLS) (*Local Area Unemployment Statistics*, 2012; *Mass Layoff Statistics Home Page*, 2013). The BLS generates local area unemployment statistics using the Current Population Survey as its source for these estimates. The BLS maintains data from initial claimants for UI associated with mass layoffs, defined as at least 50 initial claims for UI filed against a single establishment during a consecutive 5-week layoff period. Foreclosure data are from Minnesota HousingLink (*HousingLink—Research On Foreclosures In MN*, 2012). Minnesota HousingLink collects data on the number of foreclosures by working directly with individual sheriff’s offices (sheriff’s offices are responsible for the sale or auction of foreclosed property). They then used the Minnesota Department of Revenue to define the number of residential parcels in each county to compute a foreclosure rate. Further details are below regarding these variables’ operationalizations.

#### Population data

Annual population counts by county, sex, and age, are used as the denominator to calculate yearly violence-related injury rates; they are publicly available from the Surveillance, Epidemiology, and End Results Program (National Cancer Institute, n.d.).

#### Sociodemographic data

Publicly available county-level 2010 Decennial Census data and the 2010 American Community Survey are used as point-in-time estimates of potential county-level confounders of the association of economic measures and violence rates for this analysis (US Census Bureau, 2010).

The following variables are assessed: percent people of color, percent below poverty, percent minority, urban (vs. rural counties), and percent less than high school education.

### Variable Construction

#### Outcome: Violence

Primary analyses for this study use explicit codes from MHA hospital discharge data to assess child abuse, elder abuse, and IPV. However, prior research (Barata, 2011; Bhandari et al., 2006; Btoush et al., 2009; Davidov et al., 2015; Halpern et al., 2009; Lachs et al., 1997; Nannini et al., 2008; Petridou et al., 2002; Reis et al., 2009; Rosen et al., 2016; Schafer et al., 2008; Schnitzer et al., 2011; Wu et al., 2010), supports use of both explicit and proxy codes to improve measurement of violence cases. This study, therefore, includes additional analysis of child abuse, elder abuse, and IPV identified by proxy codes. To estimate rates of each violence subtype, case counts by county and year are created and merged with gender- and age-appropriate population denominators.

#### Explicit operationalization of violence

Several ICD-9 codes, E-codes, and V-codes indicate a diagnosis of child maltreatment, elder abuse, or IPV, such as ICD-9: 995.83, “child sexual abuse.” In this study, we define these diagnosis codes as “explicit” codes. The specific explicit codes used to identify each type of violence are listed in Supplemental Table 1. These codes are assigned when a medical provider ascertains, or when a patient discloses, that the injury that brought them into the hospital was due to violence.

#### Proxy operationalization of violence

Proxy ICD-9, E-Codes, and V-codes are codes that do not require an explicit diagnosis of violence, but are injury diagnoses suggestive of violence (Supplemental Tables 1 and 2). For example, a code of 362.81 for retinal hemorrhage, or bleeding in the retina, among children less than 3 years old has been previously identified to be strongly indicative of child physical abuse (Schnitzer et al., 2011). The proxy operationalizations are based on injury codes highly correlated with a “gold standard” of violence identification, using in-depth medical record review (Btoush et al., 2009; Reis et al., 2009; Schnitzer et al., 2011), predictive modeling (Barata, 2011; Reis et al., 2009), common diagnoses of known violent encounters (Bhandari et al., 2006; Davidov et al., 2015; Halpern et al., 2009; Lachs et al., 1997; Nannini et al., 2008; Petridou et al., 2002; Rosen et al., 2016; Schafer et al., 2008; Wu et al., 2010), and linkage of hospital records with known cases of violence identified in Child Protective Services (CPS) or Elder Protection Services (Lachs et al., 1997; Santaularia et al., 2021; Schnitzer et al., 2011).

### Economic Exposures

#### Economic indicator 1: Unemployment rate

The BLS provides yearly county-level unemployment rates from 2005 to 2012. The BLS estimates unemployment rates from models using monthly employment data from the Current Population Survey, the Current Employment Statistics survey, and state UI claims. The BLS unemployment rates are defined as the number of persons without employment in the reference week who had attempted to find employment during the four previous weeks, divided by the number of persons working or unemployed and looking for work in a given county (Bureau of Labor Statistics, 2015).

#### Economic indicator 2: Unemployment rate change

In addition to the unemployment rate, a yearly percentage unemployment rate change is calculated to assess whether the speed and direction of change in unemployment rates may be more predictive of violence than the unemployment rate itself (Lee et al., 2013). This measure is calculated by comparing the current year to the previous year percentage difference in unemployment.

#### Economic indicator 3: Mass-layoffs-to-workforce

A mass-layoffs-to-workforce rate is created using the total number of people laid off in the county as the numerator and BLS program data on the number of people in the work force (employed plus unemployed and looking for work) as the denominator. Mass layoffs are reported by gender. Prior evidence has suggested a gender-specific effect of mass layoffs on child abuse (Lindo et al., 2018). Therefore, gender-specific mass-layoff rates are calculated to assess gendered impacts on violence.

#### Economic indicator 4: Foreclosure rates

Foreclosure rates are calculated by Minnesota HousingLink, by dividing total number of foreclosures by number of residential parcels, staggered 1 year behind the foreclosures (*HousingLink—Research On Foreclosures In MN*, 2012). Residential parcels include homes, apartments, and farms.

### Analysis

This study includes the 87 counties in Minnesota over an 8-year period for which data are available for all economic indicators. All economic indicators are dichotomized at the mean for analysis to allow for standardization across all measures; all economic indicators are approximately normally distributed except for foreclosure rate, suggesting that dichotomizing at the mean was a reasonable approach. Sensitivity analyses exploring the impact of different cut-points were also run. Negative binomial regression models fit with generalized estimation equations to account for repeated measures over time within counties were run to estimate rate ratios for a given type of violence (e.g., IPV), comparing counties greater than or equal to the mean on each economic hardship variable to counties below the mean, averaged over the study period. Separate models were run for each of the three violence outcomes (child abuse, elder abuse, and IPV), and each of these is run first with the explicit and then separately with the proxy violence outcomes. A series of models were run for each outcome. First, crude models were run regressing the yearly count totals in a county of each operationalization of each violence outcome (e.g., explicit elder abuse, then proxy elder abuse) on each economic indicator modeled individually, with the yearly county-level population denominator for the offset (rate denominator). Second, socio-demographic-adjusted models were run adding county-level socio-demographic variables and year to each model. Third, a fully adjusted model was run for each outcome including all socio-demographic variables plus all economic indicators simultaneously. In a supplemental set of analyses, the economic variables were assessed in a 1-year lag to assess delayed effects of economic hardship on violence. Lastly, several sensitivity analyses were run to assess the robustness of findings to different categorizations of the economic hardship variables. Specifically, fully adjusted models were run using standardized continuous measures of each of the economic indicators, as well as economic indicators dichotomized at the median and top tertile, as sensitivity analyses. Results were similar to the main regression results and are thus not presented.

## Results

From 2005 to 2012, the mean county foreclosure rate was 7.4% of mortgages (*SD* = 5.5). The mean unemployment rate was 6.1% of people in the workforce (*SD* = 1.8) while the 12-month percent unemployment rate change was a 3.2% increase in unemployment rate (*SD* = 17.3). The mean explicit-identified child abuse rate was 1.0 per 1,000 (*SD* = 0.9) and the proxy-identified child abuse rate was 0.5 per 1,000 (*SD* = 0.7). The mean explicit-identified elder abuse rate was 0.1 per 1,000 (*SD* = 0.2) and the proxy-identified elder abuse rate was 16.1 per 1,000 (*SD* = 0.3). Finally, the mean explicit-identified IPV rate was 0.1 per 1,000 (*SD* = 0.2) and the proxy-identified IPV abuse rate was 11.4 per 1,000 (*SD* = 0.3) (Table 1). As reported previously (Santaularia et al., 2021), the rate of explicit defined elder abuse slightly increases over the study period (IRR_explicit_ per year: 1.03; 95% CI: 1.01–1.06), and proxy-defined elder abuse has an even stronger upward trend (IRR_proxy_ per year: 1.13; 95% CI: 1.12–1.14). The time trend for explicit child abuse and explicit IPV are both flat or slightly decreasing: Child abuse: IRR_explicit_ per year: 0.99; 95% CI: 0.98–1.00 and IPV: IRR_explicit_ per year: 0.98; 95% CI: 0.96–1.01. In contrast, child abuse and IPV proxy codes time trends are slightly increasing over time (child abuse: IRR_proxy_ per year: 1.03; 95% CI: 1.02–1.04 and IPV: IRR_proxy_ per year: 1.04; 95% CI: 1.03–1.04).

**Table 1.** Distribution of County-Level Economic Variables (*N* = 696).

Economic Measures	Mean (*SD*)	Min	Max
Foreclosure rate	7.4 (5.5)	0	33.9
Male mass-lay-offs rate	9.1 (8.4)	1	65.6
Female mass-lay-offs rate	2.3 (3.9)	0	40.1
Unemployment rate	6.1 (1.8)	2.8	14.7
12 month percent unemployment rate change	3.2 (17.3)	−23.1	71.7
Violence Measures Rate Per 1,000	Mean (*SD*)	Min	Max
Child abuse			
Explicit	1.0 (0.9)	0.0	8.5
Proxy	0.5 (0.7)	0.0	9.9
Elder abuse			
Explicit	0.1 (0.2)	0.0	1.3
Proxy	16.1 (0.3)	0.0	46.0
IPV			
Explicit	0.1 (0.2)	0.0	1.1
Proxy	11.4 (0.3)	0.4	39.0

*Note.* IPV = intimate partner violence.

In the crude models (Table 2), the foreclosure rate was most consistently and strongly related to higher rates of child abuse, elder abuse, and IPV. After foreclosure, unemployment rate showed the second most consistent, strong, and positive associations with all violence subtypes in crude models. Proxy and explicit codes show somewhat different patterns, but foreclosure and unemployment are consistently associated with both outcomes.

**Table 2.** Crude Bivariate Negative Binomial Regression With a Generalized Estimating Equation (GEE): Rate Ratio for the Association Between Each County Level Economic and Socio-Demographic Characteristics and Violence.

	Child Abuse				Elder Abuse				IPV			
	Explicit		Proxy		Explicit		Proxy		Explicit		Proxy	
	IRR	95% CI	IRR	95% CI	IRR	95% CI	IRR	95% CI	IRR	95% CI	IRR	95% CI
Foreclosure rate												
Less than 7.4	Ref	—	Ref	—	Ref	—	Ref	—	Ref	—	Ref	—
7.4 or higher	1.24	1.00–1.53	1.51	1.18–1.93	1.55	1.23–1.96	1.09	0.95–1.25	1.47	1.09–1.98	1.21	1.11–1.33
Unemployment rate												
Less than 6.1	Ref	—	Ref	—	Ref	—	Ref	—	Ref	—	Ref	—
6.1 or higher	1.22	1.01–1.47	1.21	1.00–1.48	1.37	1.07–1.75	1.35	1.22–1.50	1.14	0.93–1.41	1.24	1.13–1.36
Male mass-lay-offs rate												
Less than 9.1	Ref	—	Ref	—	Ref	—	Ref	—	Ref	—	Ref	—
9.1 or higher	1.26	0.99–1.60	1.29	1.01–1.67	0.96	0.71–1.28	1.07	0.94–1.22	1.08	0.81–1.43	1.04	0.95–1.14
Female mass-lay-offs rate												
Less than 2.3	Ref	—	Ref	—	Ref	—	Ref	—	Ref	—	Ref	—
2.3 or higher	0.93	0.79–1.08	1.16	0.86–1.56	1.04	0.83–1.31	0.95	0.85–1.07	0.89	0.64–1.22	0.96	0.88–1.05
12 month percent unemployment rate change												
Less than 3.2	Ref	—	Ref	—	Ref	—	Ref	—	Ref	—	Ref	—
3.2 or higher	1.05	0.98–1.12	1.18	0.96–1.44	0.99	0.82–1.19	1.03	0.98–1.09	0.96	0.82–1.12	0.98	0.94–1.02

**Figure 1.**
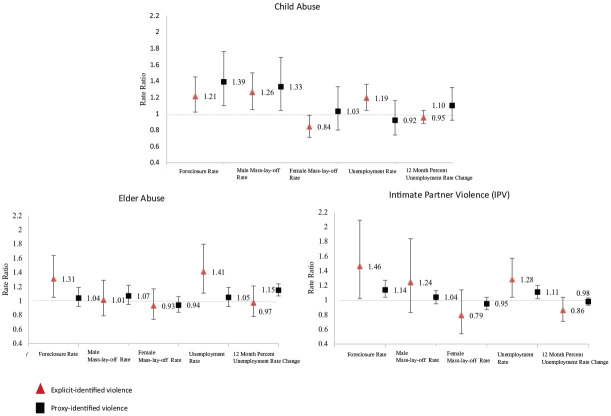
Fully adjusted* negative binomial regression with GEE: rate ratio for the association between all county level dichotomous economic and socio-demographic characteristics and explicit and proxy violence codes. *Adjusted for percent of people in poverty, percent of people of color (American Indian, Asian, Black, Two or more races, and people who are Hispanic of any race), percent of people with less than a high school education, urbancity, year, and all economic variables simultaneously.

In the sociodemographic-adjusted models (Supplemental Table 4), foreclosure and unemployment rate remained associated with increased rates of child abuse, elder abuse, and IPV. In the fully-adjusted models (Figure 1; Supplemental Table 3), mutually adjusting for all measures of economic hardship, the foreclosure rate remained most consistently and strongly related to all violence subtypes, but there is some attenuation in the associations from the crude models. For explicit child abuse, counties with greater than or equal to the mean of 7.4% of housing foreclosures have 1.21 (95% CI: 1.02–1.45) times the rate of child abuse as counties with less than 7.4% of housing foreclosures. For elder abuse this RR is 1.31 (95% CI: 1.05–1.64) and for IPV, 1.46 (95% CI: 1.02–2.09). Similar to the crude models, foreclosure rates are more strongly related to proxy than explicit child abuse codes but more weakly related to proxy than explicit elder abuse and IPV codes.

Unemployment rate remained the second most consistent risk factor for all violence subtypes in the fully adjusted models (Figure 1; Supplemental Table 3). Using explicit codes, unemployment rate had positive but varying magnitudes of association with child abuse, elder abuse, and IPV, with the weakest RR for child abuse and the strongest RR for elder abuse (Child abuse: IRR_explicit_ = 1.19; 95% CI: 1.04–1.36; Elder abuse: IRR_explicit_ = 1.41; 95% CI: 1.11–1.80; IPV: IRR_explicit_ = 1.28; 95% CI: 1.04–1.57). Using proxy codes, the relationships of unemployment with child abuse and elder abuse are close to the null. The association between unemployment rate and proxy IPV is smaller compared to explicit codes but remains, with counties with greater than or equal to 6.1% unemployment having 1.11 (95% CI: 1.02–1.20) times the rate of proxy-defined IPV compared to counties with less than 6.1% unemployment.

After adjustment for all economic and sociodemographic variables, the association between female mass-layoffs and explicit codes for child abuse suggest that counties with greater than or equal to 2.3% of female mass-layoffs have a 16% lower rate of child abuse compared to counties that had less than 2.3% of female mass-lay-offs (IRR_explicit_ = 0.84, 95% CI: 0.71–0.98), while male mass-lay-offs were associated with an increased rate (IRR_explicit_ = 1.26, 95% CI: 1.05–1.50). When using proxy child abuse codes, the association of female mass-layoffs was close to null (IRR_proxy_ = 1.03, 95% CI: 0.80–1.33), while the association with male mass layoffs was similar to that found using explicit codes (IRR_proxy_ = 1.33, 95% CI: 1.04–1.69). As in the crude models, the gendered mass-lay-off rates show no association with elder abuse and IPV defined with either explicit or proxy codes.

The lagged economic indicators generated similar results as the patterns of the fully adjusted cross-sectional models (Supplemental Table 5).

## Discussion

The goal of this paper is to determine whether county-level economic hardship predicts county rates of violence. This is done by assessing five measures of economic hardship and their contemporaneous and lagged associations with rates of both explicit and proxy injury diagnosis codes for child abuse, elder abuse, and IPV. After adjustment for sociodemographics and all other measures of economic hardship, a county’s higher foreclosure rate is the strongest and most consistent risk factor for increases in all violence subtypes. Unemployment rate is the second strongest and most consistent risk factor for violence. Lastly, there appears to be a gendered impact of mass lay-offs specific to child abuse, with male mass-lay-offs associated with increased rates and female mass-lay-offs associated with decreased rates of child abuse.

A key contribution of this study is the examination of different measures of economic hardship to assess which are most strongly related to different violence subtypes. Our study suggests that foreclosure and, to a somewhat lesser extent, unemployment rates, are the most consistent economic hardship indicators associated with higher violence of all included subtypes. Foreclosure rate may be most consistently associated with these types of violence because it is the most extreme and downstream measure of economic hardship included in this study. These findings are consistent with a systematic review of literature on child maltreatment, which reports associations between foreclosure and child maltreatment risk at the individual level (Conrad-Hiebner & Byram, 2020). In contrast, limited studies have investigated the association between foreclosure rate and IPV (Wallace et al., 2018), despite the wealth of research that supports the impact of other contextual factors on IPV (Beyer et al., 2015). No published study, to our knowledge, has previously explored the relationship between foreclosure and elder abuse. The reasons for our findings on foreclosure and violence may include disruptions to community well-being and family stress. Communities that have high foreclosure rates may subsequently have higher residential mobility rates, creating lower social cohesion and support. Low social cohesion and support increases isolation of families, thus potentially leaving them vulnerable to violence (Armstead et al., 2021). Foreclosure rates may also be the most visible measure of neighborhood economic disadvantage included in this study, due to physical deterioration of homes. Aside from the community impact of foreclosure rates, at the individual level the loss of a “home” represents not only the loss of material assets but also upends the sense of security and reliability (Kearns et al., 2000). The loss of a home also represents the loss of the primary source of American family wealth (Dunn, 2000). The family stress model (Conger et al., 1992) posits that severe economic hardship, such as experiencing home foreclosure, overwhelms all other familial resources, which may lead to more conflict and violence (Conger et al., 1994).

After foreclosure, unemployment rate is the economic hardship indicator most consistently associated with all violence subtypes in this study. There is robust literature examining these associations at the individual and ecological levels. This literature includes evidence of an increased risk of child abuse (Lee et al., 2013) and IPV (Schneider et al., 2016) in counties with higher unemployment rates. County level unemployment rate may impact violence (Agnew, 1999) through structural changes that the community undergoes during this time (Gassman-Pines et al., 2015). At the individual level, unemployment may increase future economic uncertainty and put strain on a family’s financial resources. The individual pathways between unemployment and violence may operate similarly to foreclosure and violence through the family stress model (Conger et al., 1992, 1994). However, unlike foreclosures, unemployment may be less visible and less extreme. This could explain why, in this study, the associations between unemployment rate and most of the violence measures were weaker than the associations between foreclosure rate and violence.

Another notable finding of this study is the difference in directionality of associations of male and female mass-lay-offs with child abuse. Specifically, when counties have a greater percentage of male mass-lay-off rates, there appears to be an increased rate of explicit-identified child abuse. In contrast, female mass-lay-off rates are associated with a lower rate of explicit-identified child abuse. Proxy-identified child abuse has a similar association to male mass-lay-offs as explicit-identified violence, but female mass-lay-offs’ association with proxy child abuse are close to the null. Only one prior study examined gender-specific effects of mass lay-offs. Using CPS data, researchers found that gender-specific economic shocks had opposite effects, with male mass-lay-offs showing increased risk (Lindo et al., 2018). It is possible that job losses could trigger a sense of failure relative to gender norms for the male provider. There is some evidence to suggest that when faced with status threats from job losses, male providers may act out in other ways to maintain dominance or express their masculinity, thereby increasing violent behaviors (Edwards, 2015; Fleming et al., 2015; Lindo et al., 2018). These same gender norms and expectations may lead to decreased risk of violent behavior in families in the context of female mass lay-offs, since females have been normed as the “care taker” and not the “provider” (Wingood & DiClemente, 2000).

Finally, this study adds to the literature by using proxy ICD codes to measure multiple types of violence from a statewide database of health care encounters. There was some variation in proxy and explicit violence in their association with economic hardship measures. Generally, associations of economic hardship measures with proxy-identified IPV and elder abuse were closer to the null than associations with their explicit-identified violence counterparts, as anticipated. Proxy-identified child abuse had roughly similar magnitude and directionality as compared to explicit-identified child abuse across associations with each economic hardship variable, with the exception that using proxy-identified child abuse moved the association with unemployment rate close to the null. The movement of proxy codes closer to the null could be due to a combination of factors, including the explicit codes representing selection bias toward communities with high unemployment rates (Santaularia et al., 2021). In many ways, the violence events captured through the explicit versus proxy codes may be representing different source populations, with explicit codes capturing the most visible forms of violence and proxy codes including a broader range of violence. Utilizing proxy codes for violence identification in addition to explicit codes may yield a more representative illustration of the patterning of violence. Proxy violence codes are subject to potentially less systematic bias than explicit codes. Therefore, inclusion of proxy codes may capture violence in communities where violence is not traditionally identified, that is, in whiter or wealthier communities (Sumner et al., 2015).

This ecological analysis adds to the literature as an assessment of different county level economic hardship measures and its impact on violence. Specifically, it adds context and insight to the complex and multi-level relationship that each individual has with their economy. It suggests that certain mechanisms, such as those involving social cohesion (Armstead et al., 2021) and strain (Agnew, 1999) at the community level, impact a population health outcome. It is important to emphasize that this is an ecological study, and therefore, these same associations may not be operating at the individual level.

This study is not without limitations. First, this study does not include those who experience violent events but do not go to the hospital. Therefore, this analysis may be an oversample of those with health insurance in the population (Sommers & Simon, 2017). That said, more severe or urgent injuries likely bring people in for care despite the lack of health insurance coverage (Sommers & Simon, 2017). Second, while these data are representative of violence-related injuries in hospitalized patients in Minnesota, the results may not be generalizable outside of the region. Third, none of these economic indicators fully captures economic hardship in the county. For example, unemployment rate has three main limitations (Bureau of Labor Statistics, 2015): (1) it excludes any workers who had not actively looked for work in the preceding 4 weeks before the BLS survey; (2) it does not separate out those with part-time and full-time jobs (both are considered employed—i.e., a person is considered employed if they at least have a part time job, even if they are looking for full-time employment); and (3) it does not consider underemployment, that is, if a person’s full-time job does not match their skill level and therefore their expected pay. Therefore, in several ways, the unemployment rate is an underestimate of actual unemployment. However, the study uses a wide range of economic indicators to better assess the associations between economic hardship and violence, to avoid relying on any single economic measure such as unemployment rate. Fourth, this study is ecological, which limits inference to the individual level. There is no ideal level of operationalization for economic hardship–violence associations. Ecological studies are valuable for understanding population health, as are operationalizations at other levels, which inform different aspects of how macrosocial factors are experienced as health phenomena. Fifth, this analysis does not account for spatial autocorrelation. Lastly, sixth, this is an observational study, which is subject to concerns about unmeasured confounding.

The results of this study have implications for understanding the types of economic hardship that are associated with increases or decreases in the rate of three subtypes of violence: child abuse, elder abuse, and IPV. Housing foreclosure rates were most strongly related to these outcomes, indicating that violence prevention may be particularly important when foreclosures occur. Given that foreclosure rates are a lagged or downstream measure of economic instability, there may be time for targeted interventions to occur, such as tax policies/reform programs to help prevent foreclosures and improve welfare (Sommer & Sullivan, 2018). Interventions that target childcare subsidies, elder care subsides, and mental health services could potentially help to mitigate the negative impacts on individuals, families, and communities responding to financial downturns. Future studies should examine what types of interventions are most effective in mitigating the impact of these different types of economic hardship on violence in communities.

## Supplemental Material

sj-docx-1-jiv-10.1177_08862605221118966 – Supplemental material for Economic Hardship and Violence: A Comparison of County-Level Economic Measures in the Prediction of Violence-Related Injury Click here for additional data file. Supplemental material, sj-docx-1-jiv-10.1177_08862605221118966 for Economic Hardship and Violence: A Comparison of County-Level Economic Measures in the Prediction of Violence-Related Injury by N. Jeanie Santaularia, Marizen R. Ramirez, Theresa L. Osypuk and Susan M. Mason in Journal of Interpersonal Violence

## References

[bibr1-08862605221118966] Addington W. (1999). No health insurance? It’s enough to make you sick (p. 65). The American College of Physicians-American Society of Internal Medicine. https://www.acponline.org/acp_policy/policies/no_health_insurance_scientific_research_linking_lack_of_health_coverage_to_poor_health_1999.pdf

[bibr2-08862605221118966] Agnew R. (1999). A general strain theory of community differences in crime rates. Journal of Research in Crime and Delinquency, 36(2), 123–155.

[bibr3-08862605221118966] Aizer A. (2010). The gender wage gap and domestic violence. American Economic Review, 100(4), 1847–1859. 10.1257/aer.100.4.1847 25110354 PMC4123456

[bibr4-08862605221118966] Alvira-Hammond M. Longmore M. A. Manning W. D. Giordano P. C. (2014). Gainful activity and intimate partner aggression in emerging adulthood. Emerging Adulthood, 2(2), 116–127. 10.1177/2167696813512305 25309829 PMC4191856

[bibr5-08862605221118966] Armstead T. L. Wilkins N. Nation M. (2021). Structural and social determinants of inequities in violence risk: A review of indicators. Journal of Community Psychology, 49(4), 878–906. 10.1002/jcop.22232 31421656 PMC7278040

[bibr6-08862605221118966] Ayanian J. Z. (2000). Unmet health needs of uninsured adults in the United States. JAMA, 284(16), 2061. 10.1001/jama.284.16.2061 11042754

[bibr7-08862605221118966] Barata P. (2011). The role of predictive models in identifying intimate partner violence in healthcare settings: A commentary. American Journal Preventive Medicine, 41(2), 236–237. 10.1016/j.amepre.2011.05.014 21767732

[bibr8-08862605221118966] Baumer E. P. Vélez M. B. Rosenfeld R. (2018). Bringing crime trends back into criminology: A critical assessment of the literature and a blueprint for future inquiry. Annual Review of Criminology, 1(1), 39–61. 10.1146/annurev-criminol-032317-092339

[bibr9-08862605221118966] Berger L. (2005). Income, family characteristics, and physical violence toward children. Child Abuse & Neglect, 29, 107–133. 10.1016/j.chiabu.2004.02.006 15734178

[bibr10-08862605221118966] Berger R. P. Fromkin J. B. Stutz H. Makoroff K. Scribano P. V. Feldman K. Tu L. C. Fabio A. (2011). Abusive head trauma during a time of increased unemployment: A multicenter analysis. Pediatrics, 128(4), 637–643. 10.1542/peds.2010-2185 21930535

[bibr11-08862605221118966] Beyer K. Wallis A. B. Hamberger L. K. (2015). Neighborhood environment and intimate partner violence: A systematic review. Trauma, Violence & Abuse, 16(1), 16–47. 10.1177/1524838013515758 PMC4476540 24370630

[bibr12-08862605221118966] Bhandari M. Dosanjh S. Tornetta P. Matthews D. (2006). Musculoskeletal manifestations of physical abuse after intimate partner violence. The Journal of Trauma: Injury, Infection, and Critical Care, 61(6), 1473–1479. 10.1097/01.ta.0000196419.36019.5a 17159694

[bibr13-08862605221118966] Bor J. Venkataramani A. S. Williams D. R. Tsai A. C. (2018). Police killings and their spillover effects on the mental health of black Americans: A population-based, quasi-experimental study. The Lancet (London, England), 392(10144), 302–310. 10.1016/S0140-6736(18)31130-9 29937193 PMC6376989

[bibr14-08862605221118966] Btoush R. Campbell J. C. Gebbie K. M. (2009). Care provided in visits coded for intimate partner violence in a national survey of emergency departments. Women’s Health Issues, 19(4), 253–262. 10.1016/j.whi.2009.03.004 19589474

[bibr15-08862605221118966] Bureau of Labor Statistics. (2015). How the government measures unemployment (p. 1). https://www.bls.gov/cps/cps_htgm.htm

[bibr16-08862605221118966] Cho H. Kim W. J. (2012). Intimate partner violence among Asian Americans and their use of mental health services: Comparisons with white, black, and Latino victims. Journal of Immigrant and Minority Health, 14(5), 809–815. 10.1007/s10903-012-9625-3 22527745

[bibr17-08862605221118966] Conger R. D. Conger K. J. Elder G. H. Lorenz F. O. Simons R. L. Whitbeck L. B. (1992). A family process model of economic hardship and adjustment of early adolescent boys. Child Development, 63(3), 526–541. 10.1111/j.1467-8624.1992.tb01644.x 1600820

[bibr18-08862605221118966] Conger R. D. Donnellan M. B. (2007). An interactionist perspective on the socioeconomic context of human development. Annual Review of Psychology, 58, 175–199. 10.1146/annurev.psych.58.110405.085551 16903807

[bibr19-08862605221118966] Conger R. D. Ge X. Elder G. H. Lorenz F. O. Simons R. L. (1994). Economic stress, coercive family process, and developmental problems of adolescents. Child Development, 65(2), 541–561. 10.2307/1131401 8013239

[bibr20-08862605221118966] Conrad-Hiebner A. Byram E. (2020). The temporal impact of economic insecurity on child maltreatment: A systematic review. Trauma, Violence, & Abuse, 21(1), 157–178. 10.1177/1524838018756122 29400135

[bibr21-08862605221118966] Copp J. E. Giordano P. C. Manning W. D. Longmore M. A. (2016). Couple-level economic and career concerns and intimate partner violence in young adulthood. Journal of Marriage and Family, 78(3), 744–758. 10.1111/jomf.12282 27284209 PMC4894749

[bibr22-08862605221118966] Coulton C. J. Korbin J. E. Su M. Chow J. (1995). Community level factors and child maltreatment rates. Child Development, 66(5), 1262–1276. 7555215

[bibr23-08862605221118966] Crowne S. S. Juon H. S. Ensminger M. Burrell L. McFarlane E. Duggan A. (2011). Concurrent and long-term impact of intimate partner violence on employment stability. Journal of Interpersonal Violence, 26(6), 1282–1304. 10.1177/0886260510368160 20587457

[bibr24-08862605221118966] Cunradi C. B. Caetano R. Schafer J. (2002). Socioeconomic predictors of intimate partner violence among White, Black, and Hispanic couples in the United States. Journal of Family Violence, 17(4), 377–389.

[bibr25-08862605221118966] Cutshaw C. A. Woolhandler S. Himmelstein D. U. Robertson C. (2016). Medical causes and consequences of home foreclosures. International Journal of Health Services, 46(1), 36–47. 10.1177/0020731415614249 26536913

[bibr26-08862605221118966] Davidov D. M. Larrabee H. Davis S. M. (2015). United States emergency department visits coded for intimate partner violence. Journal of Emergency Medicine, 48(1), 94–100. 10.1016/j.jemermed.2014.07.053 25282121 PMC4431640

[bibr27-08862605221118966] Deccio G. Horner W. C. Wilson D. (1994). High-risk neighborhoods and high-risk families: Replication research related to the human ecology of child maltreatment. Journal of Social Service Research, 18(34), 123–137.

[bibr28-08862605221118966] Downing J. (2016). The health effects of the foreclosure crisis and unaffordable housing: A systematic review and explanation of evidence. Social Science & Medicine, 162, 88–96. 10.1016/j.socscimed.2016.06.014 27343818

[bibr29-08862605221118966] Drake B. Pandey S. (1996). Understanding the relationship between neighborhood poverty and specific types of child maltreatment. Child Abuse and Neglect, 20(11), 1003–1018. 10.1016/0145-2134(96)00091-9 8958452

[bibr30-08862605221118966] Dunn J. R. (2000). Housing and health inequalities: Review and prospects for research. Housing Studies, 15(3), 341–366. 10.1080/02673030050009221

[bibr31-08862605221118966] Eckenrode J. Smith E. G. Mccarthy M. E. Dineen M. (2014). Income inequality and child maltreatment in the United States. Pediatrics, 133, 454–461. 10.1542/peds.2013-1707 24515511

[bibr32-08862605221118966] Edwards K. M. (2015). Intimate partner violence and the rural–urban–suburban divide. Trauma, Violence, & Abuse, 16(3), 359–373. 10.1177/1524838014557289 25477015

[bibr33-08862605221118966] Fleming P. J. Gruskin S. Rojo F. Dworkin S. L. (2015). Men’s violence against women and men are inter-related: Recommendations for simultaneous intervention. Social Science & Medicine, 146, 249–256. 10.1016/j.socscimed.2015.10.021 26482359 PMC4643362

[bibr34-08862605221118966] Fox G. L. Benson M. L. DeMaris A. A. Van Wyk J. (2002). Economic distress and intimate violence: Testing family stress and resources theories. Journal of Marriage and Family, 64(3), 793–807.

[bibr35-08862605221118966] Franklin C. A. Menaker T. A. (2014). Feminism, status inconsistency, and women’s intimate partner victimization in heterosexual relationships. Violence Against Women, 20(7), 825–845. 10.1177/1077801214543385 25031363

[bibr36-08862605221118966] Freisthler B. Bruce E. Needell B. (2007). Understanding the geospatial relationship of neighborhood characteristics and rates of maltreatment for Black, Hispanic, and White children. Social Work, 52(1), 7–16. 17388079 10.1093/sw/52.1.7

[bibr37-08862605221118966] Frioux S. Wood J. N. Fakeye O. Luan X. Localio R. Rubin D. M. (2014). Longitudinal association of county-level economic indicators and child maltreatment incidents. Maternal and Child Health Journal, 18(9), 2202–2208. 10.1007/s10995-014-1469-0 24682605 PMC4180005

[bibr38-08862605221118966] Gassman-Pines A. Gibson-Davis C. M. Ananat E. O. (2015). How economic downturns affect children’s development: An interdisciplinary perspective on pathways of influence. Child Development Perspectives, 9(4), 233–238. 10.1111/cdep.12137 31327980 PMC6641565

[bibr39-08862605221118966] Gillham B. Tanner G. Cheyne B. Freeman I. Rooney M. Lambie A. (1998). Unemployment rates, single parent density, and indices of child poverty: Their relationship to different categories of child abuse and neglect. Child Abuse & Neglect, 22(2), 79–90. 9504211 10.1016/s0145-2134(97)00134-8

[bibr40-08862605221118966] Golden S. D. Perreira K. M. Durrance C. P. (2013). Troubled times, troubled relationships: How economic resources, gender beliefs, and neighborhood disadvantage influence intimate partner violence. Journal of Interpersonal Violence, 28(10), 2134–2155. 10.1177/0886260512471083 23300198 PMC3806630

[bibr41-08862605221118966] Halpern L. R. Parry B. A. Hayward G. Peak D. Dodson T. B. (2009). A comparison of 2 protocols to detect intimate partner violence. Journal of Oral and Maxillofacial Surgery, 67, 1453–1459. 10.1016/j.joms.2009.03.003 19531417

[bibr42-08862605221118966] Heise L. L. (1998). Violence against women: An integrated, ecological framework. Violence Against Woman, 4(3), 262–290. 10.1177/1077801298004003002 12296014

[bibr43-08862605221118966] *HousingLink—Research On Foreclosures In MN*. (2012). https://www.housinglink.org/Research/ForeclosureResearch

[bibr44-08862605221118966] *ICD-9-CM—International Classification of Diseases, Ninth Revision, Clinical Modification*. (2019, March 1). https://www.cdc.gov/nchs/icd/icd9cm.htm

[bibr45-08862605221118966] Kearns A. Hiscock R. Ellaway A. MaCintyre S. (2000). “Beyond four walls”. The psycho-social benefits of home: Evidence from West Central Scotland. Housing Studies, 15(3), 387–410. 10.1080/02673030050009249

[bibr46-08862605221118966] Lachs M. S. Williams C. S. O’Brien S. Hurst L. Kossack A. Siegal A. Tinetti M. E. (1997). ED use by older victims of family violence. Annals of Emergency Medicine, 30(4), 448–454. 10.1016/S0196-0644(97)70003-9 9326859

[bibr47-08862605221118966] Lee D. Brooks-Gunn J. Mclanahan S. S. Notterman D. Garfinkel I. (2013). The Great Recession, genetic sensitivity, and maternal harsh parenting. Proceedings of the National Academy of Sciences of the United States of America, 110(34), 13780–13784. 10.1073/pnas.1312398110 23918380 PMC3752274

[bibr48-08862605221118966] Lindo J. M. Schaller J. Hansen B. (2018). Caution! Men not at work: Gender-specific labor market conditions and child maltreatment. Journal of Public Economics, 163, 77–98. 10.1016/j.jpubeco.2018.04.007

[bibr49-08862605221118966] Litton Fox G. Benson M. L . (1974). Household and neighborhood contexts of intimate partner violence. Public Health Reports, 121(4), 419–427. 10.1177/003335490612100410 PMC1525351 16827443

[bibr50-08862605221118966] Local Area Unemployment Statistics. (2012). U.S. Bureau of Labor Statistics. https://www.bls.gov/lau/

[bibr51-08862605221118966] Maguire-Jack K. Cao Y. Yoon S. (2018). Racial disparities in child maltreatment: The role of social service availability. Children and Youth Services Review, 86(October 2017), 49–55. 10.1016/j.childyouth.2018.01.014

[bibr52-08862605221118966] *Mass Layoff Statistics*. (2013). U.S. Bureau of Labor Statistics. https://www.bls.gov/mls/

[bibr53-08862605221118966] Mersky J. P. Berger L. M. Reynolds A. J. Gromoske A. N. (2009). Risk factors for child and adolescent maltreatment: A longitudinal investigation of a cohort of inner-city youth. Child Maltreatment, 14(1), 73–88. 10.1177/1077559508318399 18596199 PMC4545632

[bibr54-08862605221118966] Nannini A. Lazar J. Berg C. Barger M. Tomashek K. Cabral H. Barfield W. Kotelchuck M. (2008). Physical injuries reported on hospital visits for assault during the pregnancy-associated period. Nursing Research, 57(3), 144–149. 10.1097/01.NNR.0000319502.97864.0e 18496099

[bibr55-08862605221118966] National Cancer Institute. (n.d.). SEER data & software. Retrieved March 1, 2021, from https://seer.cancer.gov/data-software/index.html

[bibr56-08862605221118966] Pare J. (1992). Unemployment and child abuse in a rural community: A diverse relationship.

[bibr57-08862605221118966] Paxson C. Waldfogel J. (2002). Work, welfare, and child maltreatment. Journal of Labor Economics, 20(3), 435–474. 10.1086/339609

[bibr58-08862605221118966] Petridou E. Browne A. Lichter E. Dedoukou X. Alexe D. Dessypris N. (2002). What distinguishes unintentional injuries from injuries due to intimate partner violence: A study in Greek ambulatory care settings. Injury Prevention, 8, 197–201. 10.1136/ip.8.3.197 12226115 PMC1730868

[bibr59-08862605221118966] Putnam-Hornstein E. Needell B. King B. Johnson-Motoyama M. (2013). Racial and ethnic disparities: A population-based examination of risk factors for involvement with child protective services. Child Abuse & Neglect, 37(1), 33–46. 10.1016/j.chiabu.2012.08.005 23317921

[bibr60-08862605221118966] Reis B. Y. Kohane I. S. Mandl K. D. (2009). Longitudinal histories as predictors of future diagnoses of domestic abuse: Modelling study. BMJ, 339, 1–9. 10.1136/bmj.b3677 PMC2755036 19789406

[bibr61-08862605221118966] Rosen T. Clark S. Bloemen E. M. Mulcare M. R. Stern M. E. Hall J. E. Flomenbaum N. E. Lachs M. S. Eachempati S. R . (2016). Geriatric assault victims treated at U. S. trauma centers: Five-year analysis of the national trauma data bank. Injury, 47(12), 2671–2678. 10.1016/j.injury.2016.09.001 27720184 PMC5614520

[bibr62-08862605221118966] Rosenfeld R. (2009). Crime is the problem: Homicide, acquisitive crime, and economic conditions. Journal of Quantitative Criminology, 25(3), 287–306. 10.1007/s10940-009-9067-9

[bibr63-08862605221118966] Rosenfeld R. (2014). Crime and inflation in cross-national perspective. Crime and Justice, 43(1), 341–366. 10.1086/677665

[bibr64-08862605221118966] Rothman K. J. Greenland S. Lash T. L. (2008). Modern epidemiology (p. 758). Wolters Kluwer Health/Lippincott Williams & Wilkins.

[bibr65-08862605221118966] Sabina C. (2013). Individual and national level associations between economic deprivation and partner violence among college students in 31 national settings. Aggressive Behavior, 39(4), 247–256. 10.1002/ab.21479 23553507

[bibr66-08862605221118966] Santaularia N. J. Ramirez M. R. Osypuk T. L. Mason S. M. (2021). Measuring the hidden burden of violence: Use of explicit and proxy diagnosis codes for violence in Minnesota injury hospitalizations, 2004-2014. Injury Epidemiology, 8(1), 1–12. 34724989 10.1186/s40621-021-00354-6 PMC8559360

[bibr67-08862605221118966] Schafer S. D. Drach L. L. Hedberg K. Kohn M. A. (2008). Using diagnostic codes to screen for intimate partner violence in Oregon emergency departments and hospitals. Public Health Reports, 123(5), 628–635. 18828418 10.1177/003335490812300513 PMC2496936

[bibr68-08862605221118966] Schneider D. Harknett K. McLanahan S. (2016). Intimate partner violence in the great recession. Demography, 53(2), 471–505. 10.1007/s13524-016-0462-1 27003136 PMC4860387

[bibr69-08862605221118966] Schnitzer P. G. Slusher P. L. Kruse R. L. Tarleton M. M. (2011). Identification of ICD codes suggestive of child maltreatment. Child Abuse & Neglect, 35(1), 3–17. 10.1016/j.chiabu.2010.06.008 21316104

[bibr70-08862605221118966] Scott D. Tonmyr L. Fraser J. Walker S. McKenzie K. (2009). The utility and challenges of using ICD codes in child maltreatment research: A review of existing literature. Child Abuse & Neglect, 33(11), 791–808. 10.1016/j.chiabu.2009.08.005 19853915

[bibr71-08862605221118966] Shook K. Testa M. Brodkin E. Schuerman J. Mayer S. Henly J. Guthrie Annie Rosenthal C. Dugo M. Krupp K. Cunningham L. (1999). Does the loss of welfare income increase the risk of involvement with the child system? Children And Youth Services Review, 21, 781–814.

[bibr72-08862605221118966] Showalter K. (2016). Women’s employment and domestic violence: A review of the literature. Aggression and Violent Behavior, 31, 37–47. 10.1016/j.avb.2016.06.017

[bibr73-08862605221118966] Slack K. S. Lee B. J. Berger L. M. (2007). Do welfare sanctions increase child protection system involvement? A cautious answer. Social Service Review, 81(2), 207–228. 10.1086/516831

[bibr74-08862605221118966] Sommer K. Sullivan P. (2018). Implications of US tax policy for house prices, rents, and homeownership. American Economic Review, 108(2), 241–274. 10.1257/aer.20141751

[bibr75-08862605221118966] Sommers B. D. Simon K. (2017). Health insurance and emergency department use—A complex relationship. New England Journal of Medicine, 376(18), 1708–1711. 10.1056/NEJMp1614378 28467870

[bibr76-08862605221118966] Steinberg L. D. Catalano R. Dooley D. Steinberg L. D. (1981). Economic antecedents of child abuse and neglect. Child Development, 52(3), 975–985. 7285664

[bibr77-08862605221118966] Sumner S. A. Mercy J. A. Dahlberg L. L. Hillis S. D. Klevens J. Houry D. (2015). Violence in the United States: Status, challenges, and opportunities. JAMA, 314(5), 478. 10.1001/jama.2015.8371 26241599 PMC4692168

[bibr78-08862605221118966] Thurston H. Miyamoto S. (2020). Disparity in child welfare referrals from public schools: An example of Simpson’s Paradox? Child Abuse & Neglect, 102, 104397. 10.1016/j.chiabu.2020.104397 32044584

[bibr79-08862605221118966] Tobey T. McAuliff K. Rocha C. (2013). Parental employment status and symptoms of children abused during a recession. Journal of Child Sexual Abuse, 22(4), 416–428. 10.1080/10538712.2013.743951 23682767

[bibr80-08862605221118966] US Census Bureau. (2010). American Community Survey (ACS). Census.Gov. https://www.census.gov/programs-surveys/acs

[bibr81-08862605221118966] VanderEnde K. E. Sibley L. M. Cheong Y. F. Naved R. T. Yount K. M. (2015). Community economic status and intimate partner violence against women in Bangladesh: Compositional or contextual effects? Violence Against Women, 21(6), 679–699. 10.1177/1077801215576938 25845617

[bibr82-08862605221118966] Wallace D. Chamberlain A. Pfeiffer D. (2018). The relationship between foreclosures and intimate partner violence during the U.S. housing crisis. Journal of Interpersonal Violence, 36(13–14), 6247–6273. 10.1177/0886260518818431 30556475

[bibr83-08862605221118966] Wingood G. M. DiClemente R. J. (2000). Application of the theory of gender and power to examine HIV-related exposures, risk factors, and effective interventions for women. Health Education & Behavior, 27(5), 539–565. 10.1177/109019810002700502 11009126

[bibr84-08862605221118966] Wood J. N. Medina S. P. Feudtner C. Luan X. Localio R. Fieldston E. S. Rubin D. M. (2012). Local macroeconomic trends and hospital admissions for child abuse, 2000-2009. Pediatrics, 130(2), e358–e364. 10.1542/peds.2011-3755 22802600

[bibr85-08862605221118966] Wu V. Huff H. Bhandari M. (2010). Pattern of physical injury associated with intimate partner violence in women presenting to the emergency department: A systematic review and meta-analysis. Trauma, Violence, & Abuse, 11(2), 71–82. 10.1177/1524838010367503 20430799

[bibr86-08862605221118966] Young G. Gately T. (1988). Neighborhood impoverishment and child maltreatment: An analysis from the ecological perspective. Journal of Family Issues, 9(2), 240–254. 10.1177/019251388009002006

[bibr87-08862605221118966] Zuravin S. J. (1986). Residential density and urban child maltreatment: An aggregate analysis. Journal of Family Violence, 1(4), 307–322.

[bibr88-08862605221118966] Zuvekas S. H. Taliaferro G. S. (2003). Pathways to access: Health insurance, the health care delivery system, and racial/ethnic disparities, 1996–1999. Health Affairs, 22(2), 139–153. 10.1377/hlthaff.22.2.139 12674417

