# Supplementary material for: Economic Hardship and Violence: A Comparison of County-Level Economic Measures in the Prediction of Violence-Related Injury
Source: J Interpers Violence. 2022 Aug 29;38(5-6):4616–39. doi: 10.1177/08862605221118966 (PMC9900694; doi:10.1177/08862605221118966)
Supplement: sj-docx-1-jiv-10.1177_08862605221118966 – Supplemental material for Economic Hardship and Violence: A Comparison of County-Level Economic Measures in the Prediction of Violence-Related Injury [file sj-docx-1-jiv-10.1177_08862605221118966.docx]

| **Appendix 1. Explicit and Proxy ICD-9, E- and V-Codes used to Define Violence** | | | | | | | |
| --- | --- | --- | --- | --- | --- | --- | --- |
| **Child Abuse** | | | | | | | |
|  |  | **Explicit Code(s)** |  | **Proxy Code(s)** | **Exclusion Code(s)** | **Age Restriction** | **Sex** |
|  | Abuse | 995(.50-.55,.59) | Sexual abuse related codes | 54.1 , 98, 614.9, 922.4, V71.5, V71.81, | NA | < 10 | NA |
|  | Abandonment | E904.0, E968.4 | Neglect related codes | 692.7 | NA | < 2 | NA |
|  | Abuse by varies perpetrators | E 967.0-.9 |  | 994.1, E910(.2,.4,.8,.9), E960.0 | NA | < 4 | NA |
|  | Assault/homicide (struck by or against) | E960.0, E968.2 |  | 808, 860, 861, 863.8, 864,866,941,942,945,946, 960–979, E980 | NA | < 5 | NA |
|  | Assault/homicide (fire or burn) | E961, E965.6, E968(.0, .3) |  | 521, 262, E869.4, E985, V60 | NA | < 10 | NA |
|  | Assault/homicide (poisoning) | E962(.0–.2,.9) | Neglect or physical abuse related codes | 952 | NA | < 3 | NA |
|  | Assault/homicide (suffocation) | E963 |  | 800, 805, 852, 862, 863.2, 863.3, 865 | NA | < 5 | NA |
|  | Assault/homicide (cut) | E966 | Physical abuse related codes | 362.81 | NA | < 3 | NA |
|  | Assault/homicide (drowning ) | E964 |  | E965, E966, E968.2, E968.9 | NA | < 4 | NA |
|  | Assault/homicide (firearm) | E965(.0–.4) |  | 807.0, 807.1, 811, 852.2, 853 | NA | < 5 | NA |
|  | Assault/homicide (fall) | E968.1 |  | 863.1, E988 | NA | < 10 | NA |
|  | Assault/homicide (vehicle) | E968.5 |  |  |  |  |  |
|  | Assault/homicide(other ) | E965(.5–.9), E967(.0–.9), E968 (.4,.8,.9), E969 |  |  |  |  |  |
| **Elder Abuse** | | | | | | |  |
|  | Abuse | 995(.80-.85) | Abrasion | 910-919 | E928.9 | >=65 | NA |
|  | Abuse by varies perpetrators | E 967.0-.9 | Bruise | 920–924 | E928.9 | >=65 | NA |
|  | Assault/homicide (struck by or against) | E960.0, E968.2 | Burns | 940–949 | E928.9 | >=65 | NA |
|  | Assault/homicide (fire or burn) | E961, E965.6, E968(.0, .3) | Dehydration | 276.51 | E928.9 | >=65 | NA |
|  | Assault/homicide (poisoning) | E962(.0–.2,.9) | Laceration | 870–897 | E928.9 | >=65 | NA |
|  | Assault/homicide (suffocation) | E963 | Malnutrition | 262–263 | E928.9 | >=65 | NA |
|  | Assault/homicide (cut) | E966 | Pressure ulcer | 707 | E928.9 | >=65 | NA |
|  | Assault/homicide (drowning ) | E964 | Strangulation | E963 | E928.9 | >=65 | NA |
|  | Assault/homicide (firearm) | E965(.0–.4) |  |  |  |  |  |
|  | Assault/homicide (fall) | E968.1 |  |  |  |  |  |
|  | Assault/homicide (vehicle) | E968.5 |  |  |  |  |  |
|  | Assault/homicide(other ) | E965(.5–.9), E967(.0–.9), E968(.4,.8,.9), E969 |  |  |  |  |  |
|  | Rape | E960.1 |  |  |  |  |  |
| **Intimate Partner Violence** | | | | | | | |
|  | Abuse by ex-partner, ex-spouse, spouse, partner | E967.3, V611.1 | Injuries to face/head/neck | 802.0-802.9, 873.0-873.9,900.0-900.9, 910(.0-.3,.6-.9), 918.9, 920.0-921.9, 940.0- 940.5, 940.9, 941.00-941.59, 959.01, 959.09 | E810.0-E819.9 | >=16 | Female |

| **Appendix 2. Proxy Codes used to Define Violence** | | | | | | | | | |
| --- | --- | --- | --- | --- | --- | --- | --- | --- | --- |
|  |  | Code(s) | Age Restriction |  |  | Code(s) | Exclusion Code(s) | Age Restriction | Sex |
| Child sexual abuse related codes | |  |  | Child neglect or physical abuse related codes | |  |  |  |  |
|  | Genital herpes | 54.1 | < 10 |  | Skull vault fracture | 800 | N/A | < 5 | N/A |
|  | Gonococcal infection | 98 | < 10 |  | Vertebral fracture | 805 | N/A | < 5 | N/A |
|  | Pelvic inflammatory disease, unspecified | 614.9 | < 10 |  | Traumatic subarachnoid hemorrhage | 852 | N/A | < 5 | N/A |
|  | Contusion of genital organs | 922.4 | < 10 |  | Intrathoracic injury | 862 | N/A | < 5 | N/A |
|  | Observation after alleged rape | V71.5 | < 10 |  | Small intestine injury | 863.2,863.3 | N/A | < 5 | N/A |
|  | Observation for abuse/neglect | V71.81 | < 10 |  | Spleen injury | 865 | N/A | < 5 | N/A |
| Child neglect related codes | |  |  |  | Spinal cord injury | 952 | N/A | < 3 | N/A |
|  | Other severe malnutrition | 262 | <10 | Child physical abuse related codes | |  |  |  |  |
|  | Dental caries | 521 | <10 |  | Retinal hemorrhage | 362.81 | N/A | < 3 | N/A |
|  | Solar radiation dermatitis | 692.7 | < 2 |  | Rib fracture | 807.0, 807.1 | N/A | < 5 | N/A |
|  | Pelvic fracture | 808 | < 5 |  | Scapula fracture | 811 | N/A | < 5 | N/A |
|  | Traumatic pneumohemothorax | 860 | < 5 |  | Traumatic subdural hemorrhage | 852.2 | N/A | < 5 | N/A |
|  | Heart or lung injury | 861 | < 5 |  | Other/unspecified intracranial hemorrhage | 853 | N/A | < 5 | N/A |
|  | GI injury | 863.8 | < 5 |  | Stomach injury | 863.1 | N/A | < 10 | N/A |
|  | Liver injury | 864 | < 5 |  | Assault | E965, E966, E968.2 | N/A | < 4 | N/A |
|  | Kidney injury | 866 | < 5 |  | Assault, NOS | E968.9 | N/A | < 4 | N/A |
|  | Burn of head | 941 | < 5 |  | Undetermined intent, other means | E988 | N/A | < 10 | N/A |
|  | Burn of trunk | 942 | < 5 | Intimate partner violence | |  |  |  |  |
|  | Burn of leg | 945 | < 5 |  | Any burn injury to the face/head | 940.0- 940.5, 940.9, 941.00-941.59 | E810.0-E819.9 | >=16 | Female |
|  | Burn of multiple sites | 946 | < 5 |  | Any open wound injury to the face/head | 873.0-873.9, 910(.0-.3,.6-.9), 918.9, 959.01, 959.09 | E810.0-E819.9 | >=16 | Female |
|  | Poisoning by drugs/medicinals | 960–979 | < 5 |  | Fracture of face bones | 802.0-802.9 | E810.0-E819.9 | >=16 | Female |
|  | Drowning, non-fatal submersion | 994.1 | < 4 |  | Contusion to face/scalp, and neck | 920.0-921.9 | E810.0-E819.9 | >=16 | Female |
|  | Second-hand tobacco smoke | E869.4 | <10 |  | Any vessel injury to the face/head | 900.0-900.9 | E810.0-E819.9 | >=16 | Female |
|  | Swimming accident | E910.2 | < 4 | Elder Abuse | |  |  |  |  |
|  | Bathtub (near) drowning | E910.4 | < 4 |  | Abrasion | 910-919 | E928.9 | >=65 | N/A |
|  | Other (near) drowning | E910.8 | < 4 |  | Bruise | 920–924 | E928.9 | >=65 | N/A |
|  | Accidental (near) drowning, | E910.9 | < 4 |  | Burns | 940–949 | E928.9 | >=65 | N/A |
|  | Unarmed fight, brawl | E960.0 | < 4 |  | Dehydration | 276.51 | E928.9 | >=65 | N/A |
|  | Undetermined intent, poisoning | E980 | < 5 |  | Laceration | 870–897 | E928.9 | >=65 | N/A |
|  | Undetermined intent, firearm | E985 | <10 |  | Malnutrition | 262–263 | E928.9 | >=65 | N/A |
|  | Household circumstances | V60 | <10 |  | Pressure ulcer | 707 | E928.9 | >=65 | N/A |
|  |  |  |  |  | Strangulation | E963 | E928.9 | >=65 | N/A |

| **Appendix 3. Fully Adjusted^+^ Negative Binomial Regression with GEE: Rate Ratio for the Association Between All County Level Economic and Socio-demographic Characteristics and Violence** | | | | | | | | | | | | |
| --- | --- | --- | --- | --- | --- | --- | --- | --- | --- | --- | --- | --- |
|  | Child Abuse | | | | Elder Abuse | | | | IPV | | | |
|  | Explicit | | Proxy | | Explicit | | Proxy | | Explicit | | Proxy | |
|  | IRR | 95% CI | IRR | 95% CI | IRR | 95% CI | IRR | 95% CI | IRR | 95% CI | IRR | 95% CI |
| Foreclosure Rate |  |  |  |  |  |  |  |  |  |  |  |  |
| Less than 7.4 | Ref | - | Ref | - | Ref | - | Ref | - | Ref | - | Ref | - |
| 7.4 or higher | 1.21 | 1.02-1.45 | 1.39 | 1.10-1.76 | 1.31 | 1.05-1.64 | 1.04 | 0.92-1.19 | 1.46 | 1.02-2.09 | 1.14 | 1.04-1.27 |
| Unemployment Rate |  |  |  |  |  |  |  |  |  |  |  |  |
| Less than 6.1 | Ref | - | Ref | - | Ref | - | Ref | - | Ref | - | Ref | - |
| 6.1 or higher | 1.19 | 1.04-1.36 | 0.92 | 0.74-1.16 | 1.41 | 1.11-1.80 | 1.05 | 0.92-1.19 | 1.28 | 1.04-1.57 | 1.11 | 1.02-1.20 |
| Male Mass-lay-offs Rate |  |  |  |  |  |  |  |  |  |  |  |  |
| Less than 9.1 | Ref | - | Ref | - | Ref | - | Ref | - | Ref | - | Ref | - |
| 9.1 or higher | 1.26 | 1.05-1.50 | 1.33 | 1.04-1.69 | 1.01 | 0.79-1.29 | 1.07 | 0.95-1.22 | 1.24 | 0.83-1.84 | 1.04 | 0.95-1.13 |
| Female Mass-lay-offs Rate |  |  |  |  |  |  |  |  |  |  |  |  |
| Less than 2.3 | Ref | - | Ref | - | Ref | - | Ref | - | Ref | - | Ref | - |
| 2.3 or higher | 0.84 | 0.71-0.98 | 1.03 | 0.80-1.33 | 0.93 | 0.74-1.17 | 0.94 | 0.84-1.06 | 0.79 | 0.54-1.14 | 0.95 | 0.87-1.04 |
| 12 Month Percent Unemployment Rate Change |  |  |  |  |  |  |  |  |  |  |  |  |
| Less than 3.2 | Ref | - | Ref | - | Ref | - | Ref | - | Ref | - | Ref | - |
| 3.2 or higher | 0.95 | 0.88-1.04 | 1.10 | 0.92-1.32 | 0.97 | 0.78-1.21 | 1.15 | 1.07-1.24 | 0.86 | 0.71-1.04 | 0.98 | 0.93-1.03 |
| Percent of all ages in poverty |  |  |  |  |  |  |  |  |  |  |  |  |
| Less than 11.3 % | Ref | - | Ref | - | Ref | - | Ref | - | Ref | - | Ref | - |
| 11.3% or higher | 1.63 | 1.33-2.01 | 1.24 | 0.93-1.65 | 1.34 | 1.05-1.70 | 1.02 | 0.87-1.20 | 1.07 | 0.73-1.57 | 1.10 | 0.97-1.24 |
| Percent of people of color |  |  |  |  |  |  |  |  |  |  |  |  |
| Less than 9.4% | Ref | - | Ref | - | Ref | - | Ref | - | Ref | - | Ref | - |
| 9.4% or higher | 1.40 | 1.12-1.74 | 1.37 | 1.02-1.83 | 1.45 | 1.12-1.86 | 0.98 | 0.83-1.15 | 1.25 | 0.87-1.79 | 1.16 | 1.01-1.34 |
| Percent less than high school education |  |  |  |  |  |  |  |  |  |  |  |  |
| Less than 17.5% | Ref | - | Ref | - | Ref | - | Ref | - | Ref | - | Ref | - |
| 17.5% or greater | 1.00 | 0.82-1.23 | 0.86 | 0.62-1.19 | 0.66 | 0.48-0.90 | 1.18 | 0.99-1.41 | 0.79 | 0.56-1.11 | 1.06 | 0.94-1.19 |
| Urbancity |  |  |  |  |  |  |  |  |  |  |  |  |
| Rural | Ref | - | Ref | - | Ref | - | Ref | - | Ref | - | Ref | - |
| Urban | 1.00 | 0.81-1.24 | 0.89 | 0.62-1.28 | 1.02 | 0.76-1.38 | 0.92 | 0.74-1.13 | 0.96 | 0.61-1.53 | 0.94 | 0.81-1.09 |
| Year |  |  |  |  |  |  |  |  |  |  |  |  |
| Year | 0.95 | 0.92-0.97 | 1.06 | 1.02-1.10 | 0.99 | 0.95-1.04 | 1.11 | 1.09-1.13 | 0.93 | 0.89-0.98 | 1.02 | 1.01-1.04 |
| ^*People of Color includes people who are American Indian, Asian, Black, Two or more races, and people who are Hispanic of any race.^ | | | | |  |  |  |  |  |  |  |  |
| ^+ Economic variables are adjusted for simultaneously.^ |  |  |  |  |  |  |  |  |  |  |  |  |

| **Appendix 4. Sociodemographic Adjusted* Negative Binomial Regression with GEE: Rate Ratio for the Association Between Each County Level Economic and All Socio-demographic Characteristics and Violence** | | | | | | | | | | | | | | | | | | | | |
| --- | --- | --- | --- | --- | --- | --- | --- | --- | --- | --- | --- | --- | --- | --- | --- | --- | --- | --- | --- | --- |
|  | Explicit | | Proxy | | Explicit | | Proxy | | Explicit | | Proxy | | Explicit | | Proxy | | Explicit | | Proxy | |
|  | IRR | 95% CI | IRR | 95% CI | IRR | 95% CI | IRR | 95% CI | IRR | 95% CI | IRR | 95% CI | IRR | 95% CI | IRR | 95% CI | IRR | 95% CI | IRR | 95% CI |
| Child Abuse |  |  |  |  |  |  |  |  |  |  |  |  |  |  |  |  |  |  |  |  |
| Foreclosure Rate |  |  |  |  |  |  |  |  |  |  |  |  |  |  |  |  |  |  |  |  |
| Less than 7.4 | Ref | - | Ref | - |  |  |  |  |  |  |  |  |  |  |  |  |  |  |  |  |
| 7.4 or higher | 1.28 | 1.07-1.53 | 1.45 | 1.15-1.83 |  |  |  |  |  |  |  |  |  |  |  |  |  |  |  |  |
| Unemployment Rate |  |  |  |  |  |  |  |  |  |  |  |  |  |  |  |  |  |  |  |  |
| Less than 6.1 |  |  |  |  |  |  |  |  |  |  |  |  |  |  |  |  |  |  |  |  |
| 6.1 or higher |  |  |  |  | Ref | - | Ref | - |  |  |  |  |  |  |  |  |  |  |  |  |
| Male Mass-lay-offs Rate |  |  |  |  | 1.29 | 1.11-1.49 | 1.12 | 0.94-1.32 |  |  |  |  |  |  |  |  |  |  |  |  |
| Less than 9.1 |  |  |  |  |  |  |  |  | Ref | - | Ref | - |  |  |  |  |  |  |  |  |
| 9.1 or higher |  |  |  |  |  |  |  |  | 1.25 | 1.05-1.48 | 1.40 | 1.04-1.89 |  |  |  |  |  |  |  |  |
| Female Mass-lay-offs Rate |  |  |  |  |  |  |  |  |  |  |  |  |  |  |  |  |  |  |  |  |
| Less than 2.3 |  |  |  |  |  |  |  |  |  |  |  |  | Ref | - | Ref | - |  |  |  |  |
| 2.3 or higher |  |  |  |  |  |  |  |  |  |  |  |  | 0.96 | 0.83-1.12 | 1.19 | 0.87-1.62 |  |  |  |  |
| 12 Month Percent Unemployment Rate Change |  |  |  |  |  |  |  |  |  |  |  |  |  |  |  |  |  |  |  |  |
| Less than 3.2 |  |  |  |  |  |  |  |  |  |  |  |  |  |  |  |  | Ref | - | Ref | - |
| 3.2 or higher |  |  |  |  |  |  |  |  |  |  |  |  |  |  |  |  | 1.03 | 0.97-1.10 | 1.25 | 1.01-1.55 |
| Elder Abuse |  |  |  |  |  |  |  |  |  |  |  |  |  |  |  |  |  |  |  |  |
| Foreclosure Rate | Ref | - | Ref | - |  |  |  |  |  |  |  |  |  |  |  |  |  |  |  |  |
| Less than 7.4 | 1.37 | 1.10-1.72 | 1.08 | 0.95-1.22 |  |  |  |  |  |  |  |  |  |  |  |  |  |  |  |  |
| 7.4 or higher |  |  |  |  |  |  |  |  |  |  |  |  |  |  |  |  |  |  |  |  |
| Unemployment Rate |  |  |  |  |  |  |  |  |  |  |  |  |  |  |  |  |  |  |  |  |
| Less than 6.1 |  |  |  |  | Ref | - | Ref | - |  |  |  |  |  |  |  |  |  |  |  |  |
| 6.1 or higher |  |  |  |  | 1.46 | 1.15-1.86 | 1.11 | 0.97-1.26 |  |  |  |  |  |  |  |  |  |  |  |  |
| Male Mass-lay-offs Rate |  |  |  |  |  |  |  |  | Ref | - | Ref | - |  |  |  |  |  |  |  |  |
| Less than 9.1 |  |  |  |  |  |  |  |  | 1.12 | 0.85-1.47 | 1.10 | 0.97-1.25 |  |  |  |  |  |  |  |  |
| 9.1 or higher |  |  |  |  |  |  |  |  |  |  |  |  |  |  |  |  |  |  |  |  |
| Female Mass-lay-offs Rate |  |  |  |  |  |  |  |  |  |  |  |  | Ref | - | Ref | - |  |  |  |  |
| Less than 2.3 |  |  |  |  |  |  |  |  |  |  |  |  | 1.10 | 0.88-1.37 | 1.02 | 0.90-1.14 |  |  |  |  |
| 2.3 or higher |  |  |  |  |  |  |  |  |  |  |  |  |  |  |  |  |  |  |  |  |
| 12 Month Percent Unemployment Rate Change |  |  |  |  |  |  |  |  |  |  |  |  |  |  |  |  | Ref | - | Ref | - |
| Less than 3.2 |  |  |  |  |  |  |  |  |  |  |  |  |  |  |  |  | 1.03 | 0.84-1.26 | 1.19 | 1.13-1.25 |
| 3.2 or higher |  |  |  |  |  |  |  |  |  |  |  |  |  |  |  |  |  |  |  |  |
| IPV |  |  |  |  |  |  |  |  |  |  |  |  |  |  |  |  |  |  |  |  |
| Foreclosure Rate |  |  |  |  |  |  |  |  |  |  |  |  |  |  |  |  |  |  |  |  |
| Less than 7.4 | Ref | - | Ref | - |  |  |  |  |  |  |  |  |  |  |  |  |  |  |  |  |
| 7.4 or higher | 1.53 | 1.11-2.11 | 1.18 | 1.08-1.30 |  |  |  |  |  |  |  |  |  |  |  |  |  |  |  |  |
| Unemployment Rate |  |  |  |  |  |  |  |  |  |  |  |  |  |  |  |  |  |  |  |  |
| Less than 6.1 |  |  |  |  | Ref | - | Ref | - |  |  |  |  |  |  |  |  |  |  |  |  |
| 6.1 or higher |  |  |  |  | 1.38 | 1.11-1.71 | 1.15 | 1.05-1.27 |  |  |  |  |  |  |  |  |  |  |  |  |
| Male Mass-lay-offs Rate |  |  |  |  |  |  |  |  |  |  |  |  |  |  |  |  |  |  |  |  |
| Less than 9.1 |  |  |  |  |  |  |  |  | Ref | - | Ref | - |  |  |  |  |  |  |  |  |
| 9.1 or higher |  |  |  |  |  |  |  |  | 1.18 | 0.89-1.56 | 1.06 | 0.97-1.16 |  |  |  |  |  |  |  |  |
| Female Mass-lay-offs Rate |  |  |  |  |  |  |  |  |  |  |  |  |  |  |  |  |  |  |  |  |
| Less than 2.3 |  |  |  |  |  |  |  |  |  |  |  |  | Ref | - | Ref | - |  |  |  |  |
| 2.3 or higher |  |  |  |  |  |  |  |  |  |  |  |  | 0.90 | 0.67-1.19 | 1.00 | 0.92-1.09 |  |  |  |  |
| 12 Month Percent Unemployment Rate Change |  |  |  |  |  |  |  |  |  |  |  |  |  |  |  |  |  |  |  |  |
| Less than 3.2 |  |  |  |  |  |  |  |  |  |  |  |  |  |  |  |  | Ref | - | Ref | - |
| 3.2 or higher |  |  |  |  |  |  |  |  |  |  |  |  |  |  |  |  | 0.96 | 0.81-1.13 | 1.02 | 0.98-1.05 |
| ^* Adjust for percent all ages in poverty, percent people of color (includes people who are American Indian, Asian, Black, Two or more races, and people who are Hispanic of any race), percent less than highschool education, urbancity and year.^ | | | | | | | | | | | | |  |  |  |  |  |  |  |  |

| **Appendix 5. Fully Adjusted^+^ Negative Binomial Regression with GEE: Rate Ratio for the Association Between All County Level Lagged Economic and Socio-demographic Characteristics and Violence** | | | | | | | | | | | | |
| --- | --- | --- | --- | --- | --- | --- | --- | --- | --- | --- | --- | --- |
|  | Child Abuse | | | | Elder Abuse | | | | IPV | | | |
|  | Explicit | | Proxy | | Explicit | | Proxy | | Explicit | | Proxy | |
|  | IRR | 95% CI | IRR | 95% CI | IRR | 95% CI | IRR | 95% CI | IRR | 95% CI | IRR | 95% CI |
| Foreclosure Rate Lag |  |  |  |  |  |  |  |  |  |  |  |  |
| Less than 7.4 | Ref | - | Ref | - | Ref | - | Ref | - | Ref | - | Ref | - |
| 7.4 or higher | 1.16 | 0.97-1.39 | 1.36 | 1.05-1.75 | 1.54 | 1.23-1.92 | 0.97 | 0.86-1.10 | 1.43 | 0.99-2.06 | 1.15 | 1.04-1.27 |
| Unemployment Rate Lag |  |  |  |  |  |  |  |  |  |  |  |  |
| Less than 6.1 | Ref | - | Ref | - | Ref | - | Ref | - | Ref | - | Ref | - |
| 6.1 or higher | 1.23 | 1.08-1.41 | 1.04 | 0.86-1.27 | 1.19 | 0.97-1.46 | 1.09 | 0.96-1.24 | 1.37 | 1.13-1.67 | 1.07 | 0.99-1.16 |
| Male Mass-lay-offs Rate Lag |  |  |  |  |  |  |  |  |  |  |  |  |
| Less than 9.1 | Ref | - | Ref | - | Ref | - | Ref | - | Ref | - | Ref | - |
| 9.1 or higher | 1.15 | 0.95-1.38 | 1.13 | 0.95-1.34 | 1.00 | 0.75-1.32 | 1.05 | 0.93-1.18 | 1.05 | 0.73-1.51 | 1.04 | 0.95-1.13 |
| Female Mass-lay-offs Rate Lag |  |  |  |  |  |  |  |  |  |  |  |  |
| Less than 2.3 | Ref | - | Ref | - | Ref | - | Ref | - | Ref | - | Ref | - |
| 2.3 or higher | 0.82 | 0.71-0.96 | 0.95 | 0.80-1.13 | 0.86 | 0.68-1.09 | 0.99 | 0.89-1.11 | 0.85 | 0.60-1.19 | 0.95 | 0.88-1.04 |
| Percent of all ages in poverty |  |  |  |  |  |  |  |  |  |  |  |  |
| Less than 11.3 % | Ref | - | Ref | - | Ref | - | Ref | - | Ref | - | Ref | - |
| 11.3% or higher | 1.63 | 1.33-2.00 | 1.24 | 0.92-1.68 | 1.42 | 1.18-1.71 | 1.00 | 0.86-1.17 | 1.07 | 0.74-1.55 | 1.10 | 0.98-1.24 |
| Percent of people of color^*^ |  |  |  |  |  |  |  |  |  |  |  |  |
| Less than 9.4% | Ref | - | Ref | - | Ref | - | Ref | - | Ref | - | Ref | - |
| 9.4% or higher | 1.42 | 1.14-1.77 | 1.27 | 0.94-1.72 | 1.30 | 1.00-1.68 | 0.99 | 0.84-1.17 | 1.20 | 0.84-1.72 | 1.16 | 1.01-1.33 |
| Percent less than high school education |  |  |  |  |  |  |  |  |  |  |  |  |
| Less than 17.5% | Ref | - | Ref | - | Ref | - | Ref | - | Ref | - | Ref | - |
| 17.5% or greater | 0.99 | 0.82-1.21 | 0.86 | 0.63-1.17 | 0.58 | 0.44-0.77 | 1.23 | 1.04-1.46 | 0.80 | 0.57-1.12 | 1.07 | 0.95-1.21 |
| Urbancity |  |  |  |  |  |  |  |  |  |  |  |  |
| Rural | Ref | - | Ref | - | Ref | - | Ref | - | Ref | - | Ref | - |
| Urban | 0.97 | 0.78-1.21 | 0.88 | 0.62-1.24 | 0.93 | 0.69-1.24 | 0.93 | 0.76-1.14 | 0.96 | 0.60-1.54 | 0.93 | 0.80-1.08 |
| Year |  |  |  |  |  |  |  |  |  |  |  |  |
| Year | 0.94 | 0.92-0.96 | 0.99 | 0.96-1.02 | 1.00 | 0.96-1.04 | 1.05 | 1.03-1.07 | 0.92 | 0.88-0.96 | 1.02 | 1.01-1.03 |
| ^*People of Color includes people who are American Indian, Asian, Black, Two or more races, and people who are Hispanic of any race.^ | | | | | |  |  |  |  |  |  |  |
| ^+ Economic variables are adjusted for simultaneously.^ |  |  |  |  |  |  |  |  |  |  |  |  |
